# Conserved Residues Lys64 and Glu78 at the Subunit Surface of Tau Glutathione Transferase in Rice Affect Structure and Enzymatic Properties

**DOI:** 10.3390/ijms25010398

**Published:** 2023-12-28

**Authors:** Xue Yang, Zhe Zhang, Lei Wu, Meiying Yang, Siyuan Li, Jie Gao

**Affiliations:** 1 College of Life Sciences, Jilin Agricultural University, Changchun 130118, China; xyang@jlau.edu.cn (X.Y.); 20200957@mails.jlau.edu.cn (Z.Z.); lwu@jlau.edu.cn (L.W.);; 2 CAS Key Laboratory of Tropical Forest Ecology, Xishuangbanna Tropical Botanical Garden, Chinese Academy of Sciences, Menglun 666303, China

**Keywords:** glutathione transferases, hydrogen bond, ionic bond, site-directed mutagenesis, structural changes, activity changes

## Abstract

Glutathione transferases (GSTs) are a superfamily of dimeric proteins associated with the detoxification of various reactive electrophiles and responsive to a multitude of stressors. We individually substituted Lys64 and Glu78 with Ala using site-directed mutagenesis to understand the role of subunit interactions in the structure and enzymatic properties of a rice GST (OsGSTU17). The wild-type OsGSTU17 lost the conserved hydrogen bond between subunits in tau class GSTs due to conserved Tyr92 replaced with Phe92, but still exhibited high substrate activities, and thermal stability remained in its dimeric structure. The significant decrease in thermal stability and obvious changes in the structure of mutant K64A implied that conserved Lys64 might play an essential role in the structural stability of tau class GSTs. The mutant E78A, supposed to be deprived of hydrogen and salt bonds between subunits, appeared in the soluble form of dimers, even though its tertiary structure altered and stability declined dramatically. These results suggest that the hydrogen and ionic bonds provided by conserved residues are not as important for OsGSTU17 dimerization and enzymatic properties. These results further supplement our understanding of the relationship between the structure and function of GSTs and provide a theoretical basis for improving crop resistance through targeted modification of GSTs.

## 1. Introduction

Glutathione transferases (GSTs, EC 2.5.1.8), mainly in the form of dimers, are found in all classes of eukaryotic and prokaryotic organisms and catalyze a broad range of reactions involved in the addition of glutathione to substrate compounds. Typically, these are involved in nucleophilic substitution using the sulfhydryl group of reduced glutathione (GSH) to attack the electrophilic center of toxic substances, which results in the formation of hydrophilic metabolites that are readily excreted. Additionally, GSTs play a significant role in primary and secondary metabolism, stress tolerance, and cell signaling; they are also considered vigilant stress monitors capable of rapidly responding to biotic and abiotic stimuli in the surroundings [1,2,3]. Based on immunological characterizations, sequence similarity, and biochemical properties, plant GSTs are divided into 14 classes, among which four classes were previously considered to be plant-specific [3]. However, since highly homologous proteins with phi class GSTs were identified in fungi, bacteria, and protozoa, only tau, lambda, and DHAR class GSTs were recognized as plant-specific [4,5].

**Figure 1:**
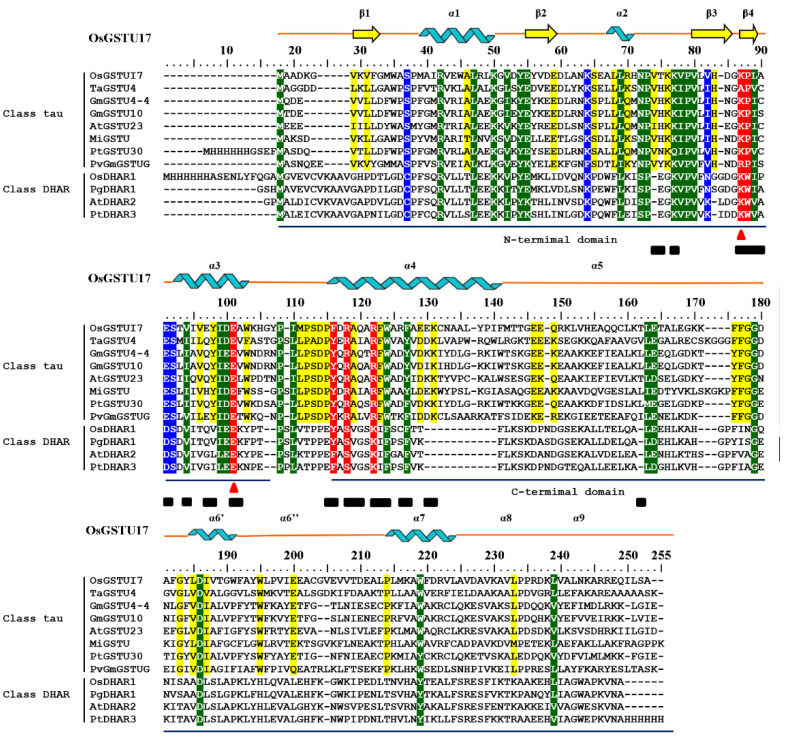
Sequence alignment of tau and DHAR class GSTs and the secondary structural element predictions of OsGSTU17. α-helices and β-strands were represented as helices and arrows, respectively. The conserved residues predicted to form hydrogen bonds and salt bridges between subunits were shaded in red. The conserved residues of the G-site were shaded in blue. The mutated sites were marked with ▲ in red. The residues buried at the interfaces of dimers were marked with ■ in black [16]. The conserved residues in all GSTs were shaded in green, whereas those among tau class GSTs were shaded in yellow. The protein sequences used in the alignment were OsGSTU17 (*Oryza sativa*, AF402804), TaGSTU4 (*Triticum tauschii*, 1GWC), GmGSTU4-4 (*Glycine max*, 2VO4), GmGSTU10 (*Glycine max*, 4CHS), AtGSTU23 (*Arabidopsis thaliana*, 6EP6), MiGSTU (*Mangifera indica*, 5G5F), PtGSTU30 (*Pinus tabulaeformis*, 5J4U), PvGmGSTUG (*Phaseolus vulgaris*, 6GHF), OsDHAR1 (*Oryza sativa*, 5D9T), PgDHAR1 (*Pennisetum glaucum,* 5EVO), AtDHAR2 (*Arabidopsis thaliana*, 5LOL), and PtDHAR3 (*Populus trichocarpa*, 2N5F).

Despite complex structural variations between distinct classes, GSTs are mainly dimeric, and each subunit contains two domains: a thioredoxin-like (βαβαββα motif) N-terminal domain and an α-helix C-terminal domain connected by a linker region (Figure 1) [6]. The highly conserved N-terminal domain encompasses conserved polar amino acid residues that exist across all cytosolic GSTs and constitute the GSH binding site (G-site), liable for triggering the GSH binding and subsequent activation of its thiol group [7,8]. The less conserved C-terminal domain forms a pocket-like hydrophobic substrate binding site (H-site), responsible for binding a variety of hydrophobic substrates, which makes it more diverse [9]. The steps of GST detoxification reactions consist of binding the reduced GSH and hydrophobic toxic substances at its G-site and H-site, respectively, to activate the thiol group of GSH, allowing for the nucleophilic attack upon the toxic substances [10]. Although each subunit has active sites of its own, the function of the enzymes can be achieved only if they form a dimer, except for classes DHAR and Lambda, which function as monomeric proteins [11]. Subunit interactions responsible for maintaining dimerization are essential for tau class GST functions.

The quaternary structure of GSTs is formed by interconnections between complementary regions in opposite subunits via hydrophobic interactions, hydrogen bonds, and salt linkages. So far, extensive investigations have been focused on the formation of quaternary structures of animal GSTs, such as the structures in pi, mu, delta, and epsilon class GSTs, in which either a lock-and-key motif located on a subunit interface or a unique subunit interface motif was identified for specific subunit recognition and dimer stabilization [12,13,14,15]. However, little is known about the underlying mechanism for maintaining the quaternary structures in plant GSTs. The only knowledge is that a significant number of conserved residues located on the interfaces of subunits that form hydrogen, ionic bonds, or hydrophobic interactions might be involved in the dimerization of the tau class GSTs [16,17]. Previous structural studies of tau class GSTs have shown that, usually, a hydrogen bond between the carboxyl group in the main chain of proline and the hydroxyl group in the side chain of tyrosine from the other subunit and salt bridges between the carboxylate group in the side chain of glutamate and guanidinium groups of two arginine in the second subunit are required to maintain the dimers [16,17,18,19]. Wang et al. found that the detriments of such interactions could lead to a complete loss of enzymatic stability and/or activities [20]. However, high enzymatic thermal stability and activities exhibited by a tau class GST OsGSTU17 in rice, which naturally lacks the hydrogen bond interaction between subunits by proline and tyrosine, were observed [21]. Therefore, the role of hydrogen bonds, salt bridges, and conserved residues on the functional tau class GSTs needs to be further elucidated. OsGSTU17 has a positive drought resistance effect in rice [22], and we took it as the research target, of which a conserved key tyrosine residue of tau class GSTs is replaced by a phenylalanine residue naturally, to understand the mechanism of the maintenance of dimers in the tau class GSTs through site-directed mutagenesis, with the aim of improving rice’s ability to resist abiotic stress through gene editing technology in the future.

## 2. Results

### 2.1. Prediction of Amino Acid Residues Involved in Subunit Interactions

**Figure 2:**
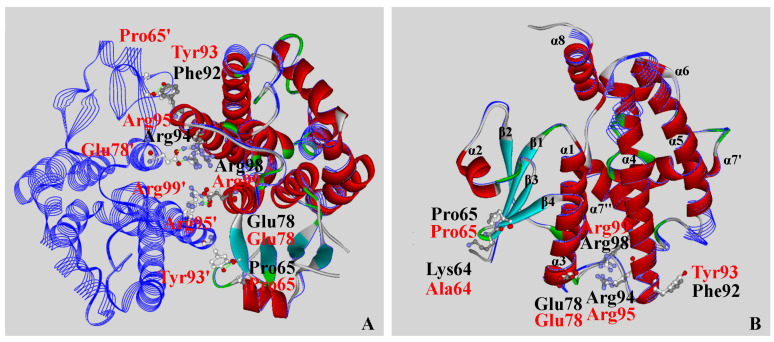
Comparison of the structure of OsGSTU17 and TaGSTU4 (PDB: 1GWC). (**A**) Overlapping of the monomeric structure of OsGSTU17 with the dimeric structure of TaGSTU4; (**B**) Comparison of spatial conformations of conserved amino acids on the surface of subunits of OsGSTU17 and TaGSTU4 proteins. The structure of OsGSTU17 was displayed in flat ribbons with α-helix shown in red and β-sheet shown in cyan. The structure of TaGSTU4 was displayed in blue line ribbons. Amino acid residues involved in hydrogen bonds and ionic bonds were displayed in balls and sticks. The carbon atoms of TaGSTU4 and OsGSTU17 were represented by white and gray balls, respectively. Nitrogen and oxygen atoms were represented by blue and red balls, respectively. Hydrogen bonds and ionic bonds were indicated by green dashes. Functional residues involved with subunit interactions in OsGSTU17 and TaGSTU4 were marked in black and red, respectively.

A significant number of conserved residues residing on the interfaces of tau class GST subunits were identified according to the sequence alignment with proteins having crystal structures in the protein data bank (PDB) (Figure 1). Three-dimensional structural alignment between the predicted tertiary structure of OsGSTU17 and structure of TaGSTU4 (PDB:1GWC) (Figure 2) revealed that the conserved amino acid residues, Pro65, Glu78, Phe92 (replacing conserved Tyr93 in TaGSTU4), Arg94, and Arg98 (alignment positions of 88, 101, 116, 118, and 122, respectively, in Figure 1) at the subunit interface of OsGSTU17 were superimposed on the residues at corresponding positions of TaGSTU4. Since these conserved amino acid residues mentioned above are responsible for formation hydrogen and salt bonds in TaGSTU4 to maintain interactions between subunits [16,17], it is speculated that these corresponding residues in OsGSTU7 might be involved in the dimerization of OsGSTU7. Interestingly, despite the natural absence of the hydrogen bond in OsGSTU17, this enzyme exhibited high stability and substrate activities [21], contrary to Wang’s observation [20], which showed that the replacement of the key residue Tyr93 with Phe led to an almost abolishment of its detoxification capability. Whether the dramatic decline in the enzymatic activities of mutant Y93F of TaGSTU4 is due to the natural replacement of a polar, positively charged Lys64 adjacent to Pro65 with a non-polar, hydrophobic Ala64 residue in TaGSTU4 needs to be clarified. Here, we explored site mutagenesis to substitute conserved residues Lys64 and Glu78 (alignment positions of 87 and 101, respectively, in Figure 1) in OsGSTU17 with Ala separately to understand the role of these amino acids in the interactions between subunits in OsGSTU17.

### 2.2. Expression and Purification of Mutant Proteins

**Figure 3:**
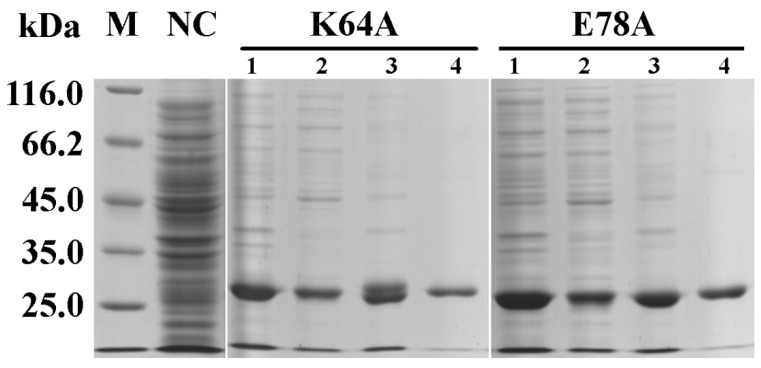
Analysis of the expression and purification of mutant proteins by SDS-PAGE. Lane M: Protein marker; Lane NC: Negative control (bacteria containing modified pET30 plasmids); Lane 1: Bacteria containing recombinant plasmids after being induced by IPTG; Lane 2: Supernatant of cell lysate by ultrasound; Lane 3: Precipitate of cell lysate by ultrasound; and Lane 4: Purified recombinant mutant proteins.

The two mutants, K64A and E78A, of OsGSTU17 were expressed in *Escherichia coli* BL21 (DE3), then purified by affinity chromatography. SDS-PAGE showed that the mutants were expressed in soluble forms (Figure 3), and both denatured proteins appeared as single bands on the gel, with a molecular weight of about 25.0 kDa, which was consistent with the expected theoretical molecular weight of the OsGSTU17 mutant monomer (Figure 3).

### 2.3. Substrate Activities of Mutant Proteins

Seven typical substrates were selected to examine the detoxification and glutathione peroxidase (GPOX) activities of the mutants and wild-type OsGSTU17 (Table 1). 7-chloro-4-nitrobenzo-2-oxa-1,3-diazole (NBD-Cl), 1-chloro-2,4-dinitrobenzene (CDNB), 4-nitrobenzyl chloride (NBC), 1,2-dichloro-4-nitrobenzene (DCNB), ethacrynic acid (ECA), and 4-nitrophenyl acetate (4-NPA) were used to detect the detoxification activities of proteins, while cumene hydroperoxide (Cum-OOH) was the usual substrate for glutathione peroxidase. Generally, compared with the wild-type OsGSTU17, the mutant proteins showed significant changes in substrate activities yet preserved their substrate specificity profiling as the same as that of the wild-type. Specifically, mutant K64A and E78A showed significant decreases in substrate activities towards NBC, retaining 3.8% and 10.1% of those of the wild-type, respectively. Towards NBD-Cl, the enzymatic activity of mutant E78A decreased by approximately 50%, and a slight decrease was observed in mutant K64A. In contrast, both mutant proteins exhibited increased activities towards CDNB, yet they were not statistically significant. The activity of mutant K64A towards Cum-OOH increased about 2.3-fold; however, the substrate activity of E78A towards Cum-OOH remained unchanged with that of the wild-type.

### 2.4. Kinetic Parameters of Mutant Proteins

The impacts of the substitution of Lys64 with Ala in OsGSTU17 on the enzymatic kinetics were explored (Table 2). Compared to the wild-type OsGSTU17, the affinities of the mutant K64A to GSH and NBD-Cl decreased significantly, with the *K*_m_ values increasing by 4- and 2-fold and the catalytic efficiencies (*k*_cat_/*K*_m_) decreasing by 80% and 46%, respectively. However, the replacement of the residue did not significantly affect the enzymatic *V*_max_ and *k*_cat_ to the substrates, indicating that the substitution of Lys64 with Ala in OsGSTU17 resulted in the disruption of the enzymatic substrate affinity, which might be the primary reason for the decline of its catalytic efficiency. The activity of mutant E78A towards the substrates was too low for the kinetic studies.

### 2.5. Structural and Thermodynamic Analysis of Mutant Proteins

Structural changes in OsGSTU17 induced by amino acid replacements were analyzed by circular dichroism (CD) spectroscopy, ANS binding assay, native PAGE (polyacrylamide gel electrophoresis), and thermal stability assessment.

**Figure 4:**
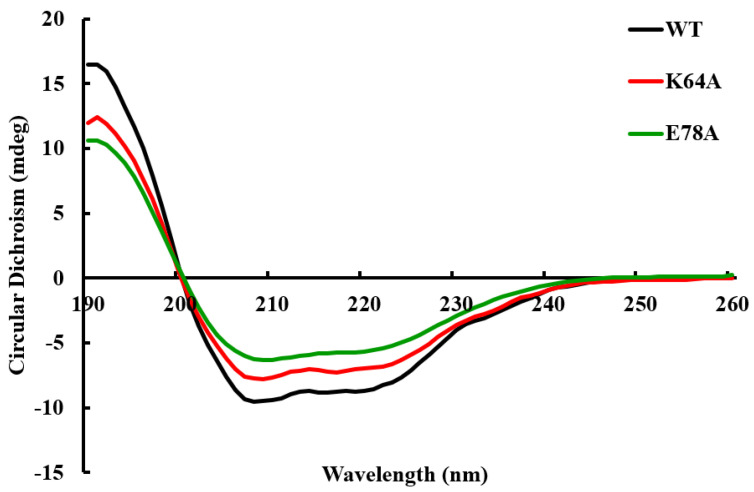
Far-UV CD spectra of the wild-type and mutants of OsGSTU17. The values of the wild-type were from our previous study [28].

**Figure 5:**
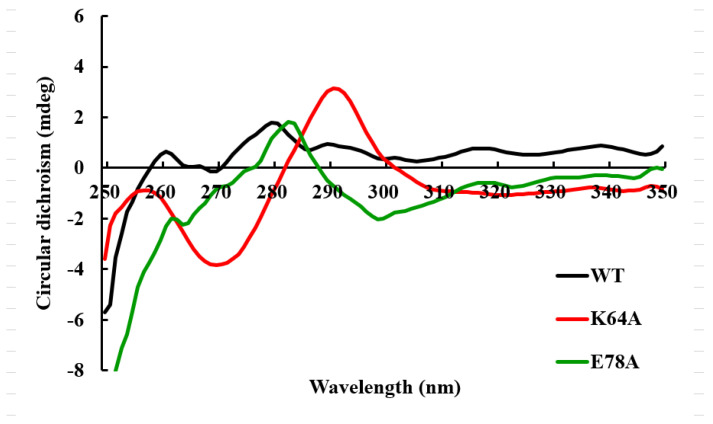
Near-UV CD spectra of the wild-type and mutants of OsGSTU17. The values of the wild-type were from our previous study [28].

CD spectra have been frequently adopted for the rapid determination of protein secondary structure and folding properties to examine the effects of mutations on protein conformation or stability [23]. Differences in far-UV CD absorbance (190–260 nm) reflect distinct variations in the secondary structures [24]. No significant change in secondary structures between mutants and wild-type OsGSTU17 was detected by far-UV CD spectroscopy analysis in this study (Figure 4). The percentages of α-helix, β-sheet, and random coils roughly calculated from the far-UV CD spectra showed that minor differences existed between the mutants and wild-type and were mainly located in the α-helix and antiparallel β-sheet (Table S1). The near-UV CD spectra (250–320 nm) reflect the properties of disulfide bonds and aromatic residues and inform the spatial conformation of aromatic residues, which were used to advise on protein tertiary structures [25,26,27]. Aromatic residues phenylalanine, tyrosine, and tryptophan exhibit distinct absorbance peaks in the wavelength regions of 250–270 nm, 270–290 nm, and 280–300 nm, respectively. The counts of residues and their conformational and environmental alterations would affect the CD signals, which would facilitate hinting at the subtle changes in protein tertiary structure caused by residue substitutions. According to the results of near-UV CD spectra (Figure 5), the mutation of the conserved amino acid residue Lys64 caused a significant change in the spatial conformation of tyrosine residues in the OsGSTU17 protein (the peak in the wavelength regions of 270–290 nm disappeared). The replacement in the conserved amino acid residue Glu78 led to the disappearance of the peak in the near-UV CD spectrum of the protein within the wavelength range of 280–300 nm, which represents the spatial conformation of tryptophan.

**Figure 6:**
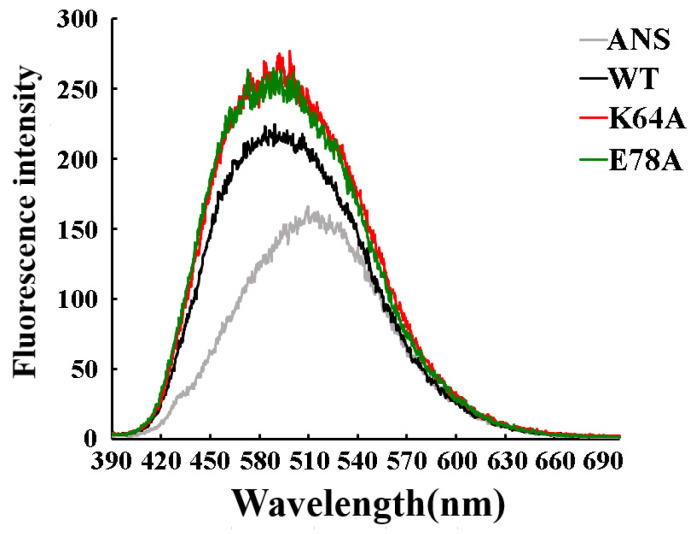
ANS fluorescence intensity of mutants and wild-type OsGSTU17. ANS represents 1-aniline-8-naphthalene sulfonate. The values of the wild-type were from our previous study [28].

ANS (1-aniline-8-naphthalene sulfonate), a fluorescent dye with low fluorescence intensity in a polar environment, when noncovalently bound with hydrophobic surfaces of proteins, its fluorescence intensity would increase significantly, accompanied by a maximum blue shift emission [29,30]. The fluorescent intensity reflects the scale of hydrophobicity of protein surfaces, which provides a clue as to what variations occurred in protein tertiary structures. The maximum fluorescent intensities of mutants K64A and E78A were almost identical (about 250 nm) but higher than that of wild-type, which indicated that the hydrophobic areas of protein OsGSTU17 were enlarged to a similar extent after the residues Lys64 and Glu78 were substituted with alanine (Figure 6).

**Figure 7:**
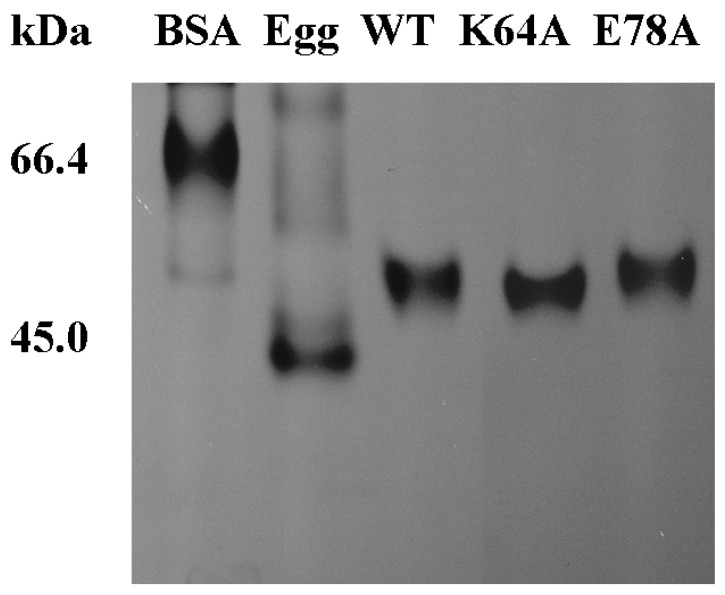
Native polyacrylamide gel electrophoresis of the purified mutant proteins and wild-type. BSA represents bovine albumin; egg represents ovalbumin.

The results of Native PAGE used for estimation of the molecular weight of mutants and wild-type could indicate whether the quaternary structure of OsGSTU17 has changed after amino acid substitution (Figure 7). The molecular weights of both the mutants K64A and E78A were similar to those of the dimeric wild-type OsGSTU17, approximately 52.0 kDa [21]. The above results, combined with the SDS-PAGE results of two denatured mutants, indicate that mutant K64A and E78A existed in the form of dimers, that is, replacing Lys64 and Glu78 with Ala will not cause the disruption of the dimeric interactions in the mutant proteins.

**Figure 8:**
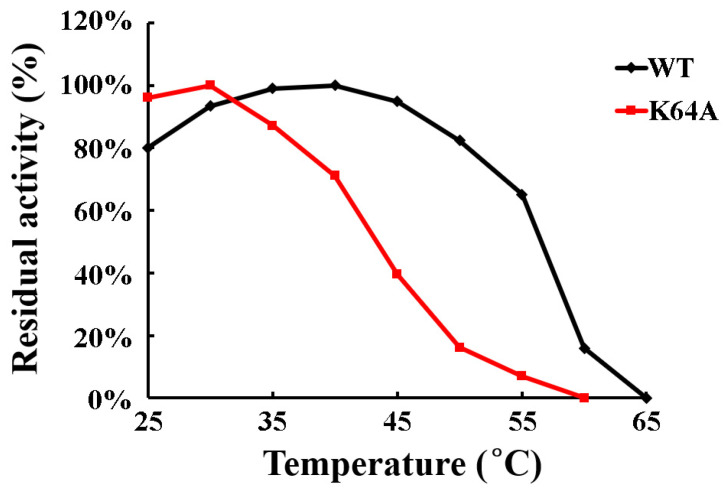
Thermodynamic stability of mutant K64A and wild-type. The values of the wild-type were from our previous study [21].

The substitutions of Lys64 and Glu78 with alanine decreased the thermal stability of OsGSTU17 to different extents, respectively (Figure 8). Compared with the wild-type, the enzyme activity of mutant E78A showed poor stability at 0 °C, with a 30% decrease in enzyme activity within 2 h at 0 °C. Therefore, reliable activity data for this enzyme could not be obtained within the range of 25 °C to 65 °C. The enzyme activity of mutant K64A remained 70% of its activity after heating at 40 °C for 15 min. Until the temperature increased to 60 °C, the enzyme activity of mutant K64A was completely abolished after being kept for 15 min. However, the wild-type OsGSTU17 still retained 80% of its maximum activity at 50 °C, while the mutant K64A only retained less than 20% activity at this temperature. The above results indicate that Glu78 is more important for the stability of OsGSTU17 than Lys64.

## 3. Discussion

Glutathione transferases (GSTs) are a large family of dimeric proteins capable of conjugating glutathione with a variety of compounds containing electrophilic centers to exert their detoxification and antioxidant functions, which rely on the active sites in each subunit as well as the dimeric structure between subunits [31]. Compared with plant GSTs, animal GST structures were more extensively studied due to their roles in drug metabolism [32,33], and it was evidenced that a unique hydrophobic lock-and-key motif and surrounding residues at the subunit interface were essential for molecular recognition in the alpha, mu, pi, epsilon, and delta classes of animal GSTs [12,13,14,15,31]. To date, though several plant GST protein structures have been resolved, which sheds light on how they bind with and catalyze substrates, less is known about their dimerization requirements, which are considered crucial for their enzymatic functions.

Tau is one class of plant GSTs that has been widely studied, especially in crops, due to its function of herbicide detoxification; however, its underlying mechanism of dimerization is still unclear. In this study, a series of conserved residues at the interface of tau class GST subunits were identified (Figure 1). Five of them, Pro65, Glu78, Tyr93, Arg95, and Arg99 (alignment positions of 88, 101, 116, 118, and 122, respectively, in Figure 1), were confirmed to be responsible for the formation of hydrogen and/or ionic bonds in TaGSTU4 and involved in the interactions between subunits [16]. The crystal structure of wheat TaGSTU4 (PDB: 1GWC) showed that a hydrogen bond was formed between the carboxyl group of the main chain of Pro65 and the hydroxyl group of the side chain of Tyr93 from another subunit [16]. In the wild-type OsGSTU17 protein, the conserved aromatic residue Tyr92 was naturally replaced with Phe92 (Figure 1), which might result in its inability to form a hydrogen bond between the subunits. However, OsGSTU17 did exert its function in the form of a dimer [21]. The conjecture that this hydrogen bond interaction was not essential for dimerization to occur in OsGSTU17 was consistent with the study of Wang et al. [20]. Also, they found that the mutant Y93F of TaGSTU4 virtually lost most of its detoxification activity, with 13% retained towards CDNB. However, the substrate specificity of wild-type OsGSTU17 (equivalent to mutant Y92F) was shown to be comparable to that of wild-type TaGSTU4. Although its activity towards CDNB was similar to that of mutant Y93F of TaGSTU4, its activity towards NBD-Cl was shown to be similar to that of the wild-type TaGSTU4. Moreover, significantly stronger activity was exhibited towards NBC by OsGSTU17 than that of TaGSTU4 (Table S2). This indicated that the effect of the substitution of Tyr92 with Phe on the enzymatic activity of OsGSTU17 was not as profound as that shown in TaGSTU4. Additionally, higher thermal stability was presented by the wild-type OsGSTU17, which retained 60% of its maximum activity at 55 °C, compared to the mutant Y93F of TaGSTU4, which was inactivated at 45 °C and 55 °C for its wild-type [20], suggesting the less important role of Tyr92 in maintaining the thermal stability of OsGSTU17. By further analysis of the protein alignment in Figure 1, the polarity of Ala64 of TaGSTU4 was opposite to that of a conserved lysine residue in the corresponding position across other tau class GSTs, including OsGSTU17, and this Ala64 was adjacent to Pro65, which formed a hydrogen bond with Tyr93 in a second subunit of TaGSTU4. We hypothesized that other differences in residues on the subunit surface may likely compensate for the loss of the hydrogen bond between Pro65 and Phe92 in OsGSTU17. We constructed a mutant K64A of OsGSTU17 so that the functional structures would be closer to those of mutant Y93F of TaGSTU4 to verify our hypothesis. Compared with the wild-type OsGSTU17, the detoxification ability of mutant K64A towards NBD-Cl and NBC decreased significantly; in particular, it lost almost its entire activity towards NBC (around a 96% reduction). And the thermodynamic stability of the mutant K64A of OsGSTU17 was shown to be more similar to that of the wild-type TaGSTU4 than to that of its mutant Y93F (Figure S1). Lys64 was located at the beginning of the β4-sheet (Figure 1), one component of the core ββα motif in the N-terminal domain, which is highly conserved across all the cytosolic GSTs, facilitating the recognition of the γ-glutamyl portion of GSH [6]. Kinetic analysis showed that the substitution of Lys64 with Ala significantly disrupted the affinity of OsGSTU17 to GSH (Table 2). Meanwhile, the near-UV CD spectroscopy results showed significant changes in the spatial conformation of aromatic amino acids in the mutant K64A (Figure 5), but no aromatic amino acids were found next to Lys64 in the structure of OsGSTU17. In the primary structure, the closest aromatic amino acid to Lys64, tyrosine (Tyr75), was located in the α3-helix (Figure 1). Since α3-helix is adjacent to the β4-sheet, the substitution of the Lys64 located at the starting position of the β4-sheet may cause a shift in the spatial conformation of the α3-helix, which explains the disappearance of the tyrosine peak in the near-UV CD spectrum of mutant K64A. Meanwhile, due to this substitution, the spatial conformation of the α3-helix in the core ββα motif has changed, suggesting that the interaction force between amino acids on the subunit surface of mutant K64A has decreased. When external energy (increasing temperature) is applied to wild-type and mutant K64A, the structural instability of mutant K64A leads to a decrease in activity, resulting in lower thermal stability than wild-type. But this change did not result in the depolymerization of the protein. However, the effects of the substitution of Lys64 residue on the glutathione peroxidase (GPOX) activity of OsGSTU17 were shown to be contrary to those on its detoxification capacity. The activity of mutant K64A towards Cum-OOH were increased about 2-fold (Table 1). This suggested that different catalytic mechanisms may be adopted by OsGSTU17 towards Cum-OOH and hydrophobic toxic substances, respectively, which was consistent with our previous finding that variations in residues at the H-site could result in inverse consequences in the detoxification capabilities and GPOX activities of OsGSTU17 [8].

Salt bridges have been proven to play an important role in protein folding, stability, and catalysis [34,35,36]. In addition to hydrogen bonds, the salt bridge interactions between glutamate of one subunit and two arginines of the other subunit were observed in both dimeric crystal structures of GmGSTU4-4 [17] and TaGSTU4 [16]. The three conserved residues, according to the alignment sites, Glu101, Arg118, and Arg122 in Figure 1, involved in the salt bridge formation were also identified in OsGSTU17. These residues were superimposed on the similar spatial conformation of the crystal structure of TaGSTU4 (Figure 2), suggesting that there may be similar electrostatic interactions between Glu78, Arg94, and Arg98 in OsGSTU17. When residues involved in ionic bonds were replaced with non-polar amino acids, TaGSTU4 protein either existed in insoluble form or abolished its entire activity. However, when these residues were substituted with an amino acid of the same polarity, the protein restored its dimeric form expressed in soluble form, and except for Cum-OOH, their activities towards other substrates were significantly reduced or lost entirely [20]. This indicated that the ionic bonds were critical to the folding, stability, and substrate activity of TaGSTU4. However, this study showed the ionic bonds seemed to be less important for OsGSTU17. The conserved amino acid residue Glu78 is located near the end of the α3-helix, and the substitution of it with alanine may shorten the length of the α3-helix (the proportion of α-helix decreases, Table S1, and the degree of reduction is greater than that of mutant K64A), thereby affecting the spatial conformation of tryptophan (Trp80), located one amino acid away from Glu78. This structural change resulted in the disappearance of the peak of mutant E78A in the near-UV CD spectrum at 280–300 nm. At the same time, the shortening of the α3-helix may lead to a decrease in the intermolecular forces between subunits, resulting in poorer stability of mutant E78A under low-temperature conditions, and its impact on protein stability is greater than that of mutant K64A. Even though the mutant E78A of OsGSTU17 exhibited poor thermal stability, it existed in soluble form, which differs from the mutant E78A of TaGSTU4. Meanwhile, compared with the wild-type, the mutant E78A of OsGSTU17 reserved its substrate specificity and slightly increased its activity towards CDNB. Thus, we speculated that these salt bridges were not a necessary force for maintaining the dimer of OsGSTU17, but they might facilitate the enhancement of the protein’s thermal stability [37]. When the Glu78 was replaced by Ala, we found that the protein remained in dimer form even though it had lost both hydrogen and ionic bonds (Figure 3 and Figure 7), which suggested that there were likely other interactions that existed in OsGSTU17 to assist in the formation of dimers. From the alignment of protein sequences, DHAR class GSTs also contained the conserved structural basis for the hydrogen bond and/or salt bridges (Figure 1), but DHAR class GSTs function in the form of monomers, which indicates that the hydrogen bond or salt bond might not be the main force to form dimers, and there might be other structural basis or forces that exist for assisting in the maintenance of dimers of tau class GSTs. It was speculated that hydrophobic interactions might be the primary force for the dimerization of OsGSTU17. Numerous studies have suggested that hydrophobic interactions were stronger than salt bridges in protein folding, and unique hydrophobic interactions existed between subunits of GSTs [12,13,14,15,31,34,36,38,39,40]. According to the sequence analysis by Thom et al. [16], the conserved residues residing at the dimer interface account for a large proportion of the conserved residues in the tau class GSTs. However, whether these amino acids interact with each other and whether these interactions contribute to protein dimerization needs to be further explored.

## 4. Materials and Methods

### 4.1. Alignment of Protein Sequences

The protein sequences of tau and DHAR class GSTs with crystal structure information were aligned with OsGSTU17 using BioEdit 7.0.5.3 [41]. The sequences involved include OsGSTU17 (*Oryza sativa*, AF402804), TaGSTU4 (*Triticum tauschii*, 1GWC), GmGSTU4-4 (*Glycine max*, 2VO4), GmGSTU10 (*Glycine max*, 4CHS), AtGSTU23 (*Arabidopsis thaliana*, 6EP6), MiGSTU (*Mangifera indica*, 5G5F), PtGSTU30 (*Pinus tabulaeformis*, 5J4U), PvGmGSTUG (*Phaseolus vulgaris*, 6GHF), OsDHAR1 (*Oryza sativa*, 5D9T), PgDHAR1 (*Pennisetum glaucum*, 5EVO), AtDHAR2 (*Arabidopsis thaliana*, 5LOL), and PtDHAR3 (*Populus trichocarpa*, 2N5F).

### 4.2. Three-Dimensional Structural Prediction of OsGSTU17 Protein

Taking 2VO4 (Protein Data Bank, PDB) as the template, the three-dimensional structure model of OsGSTU17 was constructed by homologous modeling using Insight II 2005 (Accelrys Software Inc., San Diego, CA, USA). The three-dimensional structural comparison and analysis of OsGSTU17 and TaGSTU4 were completed using Accelrys Discovery Studio version 1.6 (Accelrys Software Inc., San Diego, CA, USA).

### 4.3. Construction of Mutants

The mutant sequences of OsGSTU17 were generated by overlap extension using two-round polymerase chain reactions (PCR) described by Ho et al. [42]. Briefly, in the first round of PCR, two overlapped fragments (left and right) were obtained by two separate PCR reactions using the plasmid containing the cDNA of OsGSTU17 as the template. The left fragment containing the mutation in the 3′ end was obtained by PCR using R and Ex1 as primers; the right fragment was obtained by PCR using F and Ex2 as primers. In the second round of PCR, both the purified left and right overlapped fragments were used as the template, and primers Ex1 and Ex2 with restriction sites were used to assemble the sequences containing mutations of interest. The two rounds of PCRs were performed using *Pyrobest*^®^ DNA Polymerase (TaKaRa, Dalian, China). The primer sequences are shown in Table S3. The purified PCR product was digested by *Eco*RI and *Hind*III (New England Biolabs Ltd., Beijing, China) and subcloned into the modified prokaryotic expression vector pET30a, which carried an 18-nucleotide fragment encoding 6×His-tag between the start codon and multiple cloning site (MCS) to avoid the influence of non-OsGSTU17 protein amino acids on its folding [21]. The recombinant plasmids ΔpET30a/Mutant were transformed into *E. coli* BL21 (DE3). The mutated sequences were confirmed by Sanger sequencing (Beijing Genomics Institute, Shenzhen, China).

### 4.4. Expression and Purification of Mutant Recombinant Proteins

The expression, purification, and desalination of proteins were carried out according to the description of Yang et al. [21]. The overnight culture of *E. coli* BL21 (DE3) containing the recombinant plasmids ΔpET30a/Mutant was diluted at 1:100 and cultured to an OD value of 0.6, then the inducer of isopropylthio-β-D-galactopyranoside (IPTG) was added to a final concentration of 0.1 mM. The bacteria were harvested after a continuous culture for 12 h at 37 °C and then resuspended with binding buffer (20 mM Na_3_PO_4_, 0.5 M NaCl, 20 mM imidazole, pH = 7.4). The resuspended solution mixture was sonicated at 0 °C for 10 min with a 5 min interval. The supernatant containing the target protein was then loaded onto a pre-equilibrated Ni Sepharose High Performance column (Amersham Pharmacia Biotech, GE Healthcare, Chicago, IL, USA). The precipitate was dissolved in binding buffer and then detected with 12% SDS—polypropylene gel electrophoresis (SDS-PAGE). The mutant proteins with 6×His-tag were supposed to be bound to the nickel ion chelated on Sepharose, then eluted with elution buffer (20 mM Na_3_PO_4_, 0.5 M NaCl, 500 mM imidazole, pH = 7.4) and collected. The purified target proteins were then loaded onto a PD-10 desalting column (Amersham Pharmacia Biotech, GE Healthcare, Chicago, IL, USA) for buffer exchange. Finally, the mutant proteins were dissolved in 10 mM Tris-HCl buffer (pH = 7.4). The purity of the purified protein was detected by SDS-PAGE, and the concentration of the protein was determined by its absorbance value at 280 nm, measured by a Thermo Scientific™ Evolution™ 300 (Waltham, MA, USA) ultraviolet spectrophotometer.

### 4.5. Detection of Enzymatic Properties

Substrates NBD-Cl, CDNB, NBC, DCNB, ECA, 4-NPA, and Cum-OOH (Merck, Germany), which are typical substrates for detecting detoxification and GPOX activities of tau class GSTs, were used to investigate the enzyme activities of wild-type and mutants. The activity towards NBD-Cl was determined according to the method described by Ricci et al. [43], the activity towards CDNB, NBC, DCNB, and ECA was determined according to the methods described by Habig et al. [44], and the activity towards Cum-OOH was determined according to the method described by Edwards and Dixon [45]. Taking NBD-Cl and GSH as substrates (Merck, Germany), the kinetic parameters of mutant enzymes were monitored according to the description of Yang et al. [21]. The *K*_m_ and *V*_max_ values for GSH were obtained by fixing the NBD-Cl concentration at 1.0 mM and detecting the enzyme activity with a GSH concentration in the range of 0.2 to 1.0 mM. The *K*_m_ and *V*_max_ values for NBD-Cl were obtained by fixing the GSH concentration at 1.0 mM and detecting the enzyme activity with NBD-Cl concentrations ranging from 0.2 to 1.0 mM. Non-linear regression analysis of Graphpad Prism 10.0 (Graphpad Software Inc., San Diego, CA, USA) was applied to the data to obtain the kinetic parameters of mutants. The experimental data were represented as the average of three independent experiments.

### 4.6. Detection of Protein Structure Changes

The circular dichroism (CD) spectra of near- and far-UV absorbance of the mutants and wild-type OsGSYU17 were measured by Chirascan-plus Dynamic Multimode Spectroscopy (DMS) (Applied Photophysics Inc., Leatherhead, UK). The final absorbent values were determined by subtracting the buffer-blank spectrum from that acquired for the protein samples, and the reported spectra were presented as the average of three scans for each sample. The near-UV CD data were obtained by dissolving the proteins in elution buffer without imidazole (20 mM Na_3_PO_4_, 0.5 M NaCl, pH 7.4) to a concentration of 0.91 mg/mL in a 0.5 mm pathlength cell at 25 °C. The far-UV CD data of proteins were obtained by dissolving the proteins in 10 mM Tris-HCl (pH 7.4) buffer to a concentration of 0.24 mg/mL in a 10 mm pathlength cell at 25 °C.

For the ANS binding assay, the fluorescence intensity of ANS was detected by the HITACHI F-4500 FL Spectrophotometer with a 1 mL reaction mixture (PBS buffer, pH = 7.4) containing 200 μM ANS and enzymes with a final concentration of 0.05 mg/mL. The parameters of excitation wavelength, receiving range of emission spectrum, scanning speed, excitation and emission slit, and PMT voltage were determined according to Wang et al. [20]. Triplicate scanning data of both the blank and sample were recorded, respectively. The reported spectra were represented as the average of three independent experiments.

Native PAGE was performed according to the method described by Nijtmans et al. [46]. To remove the monomers, dimers, and polymerization initiator from the gel and improve its resolution, pre-electrophoresis of the prepared gel was performed for 30–60 min before the samples were loaded. In the process of electrophoresis, the voltage was set between 100 and 200 V, and the ambient temperature was kept at 0–4 °C to avoid protein denaturation due to heat generated by high voltage.

The thermal stability curves of mutants were plotted based on the activity of the mutant towards CDNB. The activities towards the substrate were monitored after the enzyme was incubated at 25 to 55 °C for 15 min at intervals of 5 °C, respectively. The experimental results represented the average of three independent experiments.

## 5. Conclusions

In conclusion, the five conserved residues, Pro65, Glu78, Tyr93, Arg95, and Arg99, identified in TaGSTU4 as responsible for the formation of hydrogen bonds and ionic bonds between subunits, played an important role in its folding and enzymatic activities. However, the corresponding residues identified in OsGSTU17 displayed functional differences compared to the residues in TaGSTU4. The conserved residue Glu78 in OsGSTU17 was revealed to be essential for the protein’s thermal stability, while the hydrogen bonds formed by it on the subunit surface did not play a crucial role in protein dimerization. But interestingly, the hydrogen bonds formed by Glu78 were indispensable in the dimerization of the TaGSTU4 protein. The conserved Lys64 residue at the subunit interface did not participate in the formation of hydrogen bonds and had no effect on protein dimerization. However, the change in this residue was not conducive to the thermal stability of the OsGSTU17 protein or its binding with GSH. Therefore, a question is raised: “Why does TaGSTU4 still retain alanine at this position during evolution?” The relationship between amino acids and structure still need further research and exploration. It is inferred that the interactions between other amino acids on the surface of protein subunits, such as hydrophobic interactions, might be the primary force for maintaining protein dimerization.

## Figures and Tables

**Table 1:** Specific activities of K64A and E78A mutants and wild-type.

Substrates	Specific Activity (μmol min^−1^ mg^−1^)		
	WT [21]	K64A	E78A
NBD-Cl	0.203 ± 0.006	0.180 ± 0.001	0.087 ± 0.001
CDNB	0.113 ± 0.019	0.158 ± 0.003	0.138 ± 0.007
NBC	1.153 ± 0.046	0.044 ± 0.013	0.117 ± 0.005
Cum-OOH	0.013 ± 0.002	0.030 ± 0.001	0.014 ± 0.001
DCNB	ND	ND	ND
4-NPA	ND	ND	ND
ECA	ND	ND	ND

Note: The values shown are means ± S.D., calculated from three replicates. The values for the wild-type were obtained from our previous study [21]. NBD-Cl represents 7-chloro-4-nitrobenzo-2-oxa-1,3-diazole; CDNB represents 1-chloro-2,4-dinitrobenzene; NBC represents 4-nitrobenzyl chloride; Cum-OOH represents cumene hydroperoxide; DCNB represents 1,2-dichloro-4-nitrobenzene; 4-NPA represents 4-nitrophenyl acetate; and ECA represents ethacrynic acid.

**Table 2:** Kinetic analysis of mutant K64A and wild-type.

	GSH				NBD-Cl			
	*K*_m_	*V*_max_	*k*_cat_	*k*_cat_/*K*_m_	*K*_m_	*V*_max_	*k*_cat_	*k*_cat_/*K*_m_
	(mM)	(μM min^−1^ mg^−1^)	(S^−1^)	(mM^−1^S^−1^)	(mM)	(μM min^−1^ mg^−1^)	(S^−1^)	(mM^−1^S^−1^)
WT [21]	0.058 ± 0.006	0.225 ± 0.011	0.264	4.552	0.324 ± 0.016	0.219 ± 0.006	0.257	0.793
K64A	0.233 ± 0.001	0.226 ± 0.001	0.171	0.924	0.507 ± 0.025	0.287 ± 0.010	0.217	0.428

Note: The values shown are means ± S.D., calculated from three replicates. The values for the wild-type were obtained from our previous study [21].

## Data Availability

Data is contained within the article or Supplementary Material.

## Supplementary Materials

The supporting information can be downloaded at: https://www.mdpi.com/article/10.3390/ijms25010398/s1.

## Abbreviations

GSTs	Glutathione transferases
NBD-Cl	7-chloro-4-nitrobenzo-2-oxa-1,3-diazole
CDNB	1-chloro-2,4-dinitrobenzene
NBC	4-nitrobenzyl chloride
Cum-OOH	Cumene hydroperoxide
DCNB	1,2-dichloro-4-nitrobenzene
4-NPA	4-nitrophenyl acetate
ECA	Ethacrynic Acid
ANS	1-aniline-8-naphthalene sulfonate
CD	Circular Dichroism
