# Supplementary material for: Conserved Residues Lys64 and Glu78 at the Subunit Surface of Tau Glutathione Transferase in Rice Affect Structure and Enzymatic Properties
Source: Int J Mol Sci. 2023 Dec 28;25(1):398. doi: 10.3390/ijms25010398 (PMC10778600; doi:10.3390/ijms25010398)
Supplement: Supplementary file 1 [file ijms-25-00398-s001.zip › Figure S1.pdf]

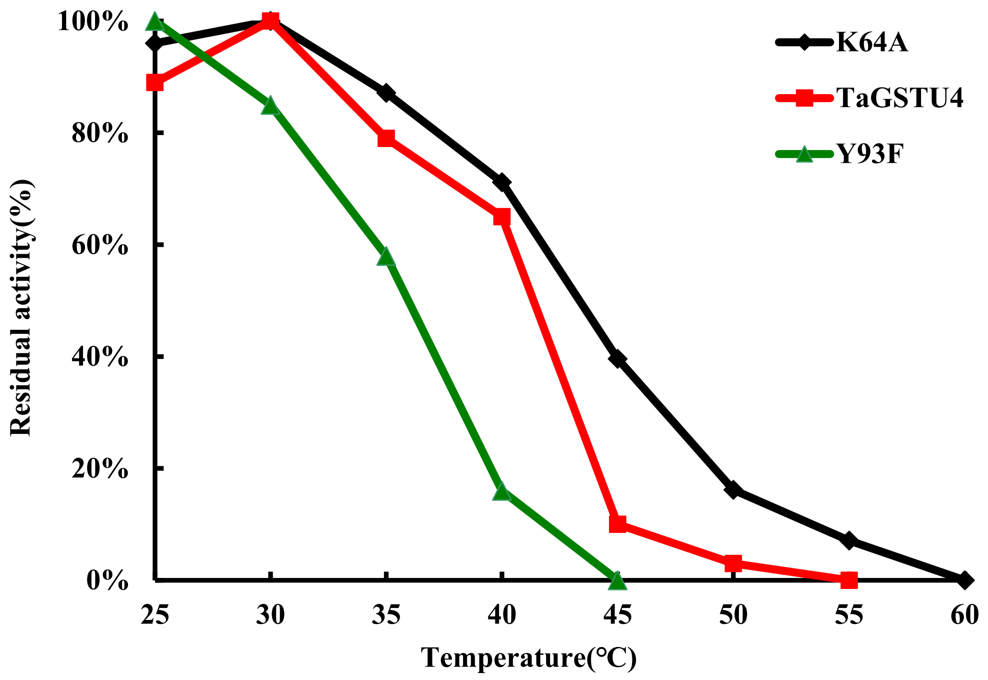

Figure S1. Thermodynamic stability of mutant K64A of wild-type OsGSTU17, wild-type TaGSTU4 and its mutant Y93F. The values of TaGSTU4 and its mutant Y93F were obtained from Wang *et al* [20].
