# Supplementary material for: Conserved Residues Lys64 and Glu78 at the Subunit Surface of Tau Glutathione Transferase in Rice Affect Structure and Enzymatic Properties
Source: Int J Mol Sci. 2023 Dec 28;25(1):398. doi: 10.3390/ijms25010398 (PMC10778600; doi:10.3390/ijms25010398)
Supplement: Supplementary file 1 [file ijms-25-00398-s001.zip › Table S1.pdf]

Table S1. Percentage of secondary structures of different types in mutant K64A and E78A and wild type of OsGSTU17

Protein	Helix	Antiparallel	Parallel	Beta-Turn	Rndm. Coil
WT[28]	28.8%	14.7%	9.0%	18.1%	32.3%
K64A	25.6%	19.3%	9.9%	18.7%	34.9%
E78A	23.4%	22.6%	10.5%	19.1%	36.7%

Note: Helix represents α-helix; Antiparallel represents antiparallel β-sheet; Parallel represents β-sheet; Beta-Turn represents β-turn; Rndm. Coil represents random coil. The values for the wild type were obtained from our previous study [28].
