# Supplementary material for: Conserved Residues Lys64 and Glu78 at the Subunit Surface of Tau Glutathione Transferase in Rice Affect Structure and Enzymatic Properties
Source: Int J Mol Sci. 2023 Dec 28;25(1):398. doi: 10.3390/ijms25010398 (PMC10778600; doi:10.3390/ijms25010398)
Supplement: Supplementary file 1 [file ijms-25-00398-s001.zip › Table S2.pdf]

Table S2. Substrate activities of wild-type OsGSTU17, wild-type TaGSTU4 and its mutant Y93F

Substrates	Enzymatic activities (μmol min-1 mg-1)		
	**OsGSTU17 [21]**	**TaGSTU4 [20]**	**Y93F of TaGSTU4 [20]**
NBD-Cl	0.203 ± 0.006	0.19 ± 0.039	ND
CDNB	0.113 ± 0.019	1.08 ± 0.009	0.14 ± 0.028
NBC	1.153 ± 0.046	0.39 ± 0.012	ND
Cum-OOH	0.013 ± 0.002	0.15 ± 0.005	0.14 ± 0.003

Note: The values were shown as means ± S.D., calculated from triplicates. ND represents Not Detected. The values of OsGSTU17 were obtained from our previous study [21]. The values of TaGSTU4 were obtained from Wang et al [20].
