# Supplementary material for: Conserved Residues Lys64 and Glu78 at the Subunit Surface of Tau Glutathione Transferase in Rice Affect Structure and Enzymatic Properties
Source: Int J Mol Sci. 2023 Dec 28;25(1):398. doi: 10.3390/ijms25010398 (PMC10778600; doi:10.3390/ijms25010398)
Supplement: Supplementary file 1 [file ijms-25-00398-s001.zip › Table S3.pdf]

**Table S3.** Primers used for construction of OsGSTU17 mutants

| Mutants | Primer names | Primer sequences (5'-3') |
|---|---|---|
| K64A | K64A-R | GAC GGT GGA CTC GGC GAG AGG CGC GCC GTC GTG GAC CAG CAC GG |
| | K64A-F | CCT CTC GCC GAG TCC ACC GTC |
| E78A | E78A-R | GGG GTA GCC GTG CTT CCA GGC CGC GTC GAT GTA CTC GAC GAT GA |
| | E78A-F | GCC TGG AAG CAC GGC TAC CCC |
| | OsGSTU17-Ex1 | T*GA ATT C*AT GGC AGC AGA CAA GGG AG |
| | OsGSTU17-Ex2 | T*AA GCT T*TT ACT ACG CCG AGA GGA TCT G |

Note: F is short for forward primer; R is short for reverse primer. Mutation sites were underlined. Sites of restriction endonuclease *Eco*R I and *Hind* III were marked with italic.
